# Dexmedetomidine and sufentanil combination versus sufentanil alone for postoperative intravenous patient-controlled analgesia: a systematic review and meta-analysis of randomized controlled trials

**DOI:** 10.1186/s12871-019-0756-0

**Published:** 2019-05-18

**Authors:** Miaomiao Feng, Xuhui Chen, Tongtong Liu, Chuanhan Zhang, Li Wan, Wenlong Yao

**Affiliations:** Department of Anesthesiology, Tongji Hospital, Tongji Medical College, Huazhong University of Science and Technology, Wuhan, 430030 China

**Keywords:** Dexmedetomidine, Sufentanil, Patient-controlled analgesia

## Abstract

**Background:**

Previous studies have demonstrated that dexmedetomidine improves the quality of postoperative analgesia. In the present study, we performed a meta-analysis of randomized controlled trials to quantify the effect of dexmedetomidine as an adjuvant to sufentanil for postoperative patient-controlled analgesia (PCA).

**Methods:**

PubMed, Embase, the Cochrane Library, and Web of Science were systematically searched for randomized controlled trials in which dexmedetomidine was used as an adjuvant for PCA with sufentanil. In the retrieved studies, we quantitatively analyzed pain intensity, sufentanil consumption, and drug-related side effects.

**Results:**

Nine studies with 907 patients were included in this meta-analysis. Compared with sufentanil alone, dexmedetomidine-sufentanil for postoperative intravenous PCA reduced pain intensity at 24 h (mean difference (MD) = − 0.70points; 95% confidence interval (CI): − 1.01, − 0.39; *P* < 0.00001) and 48 h postoperatively (MD = -0.61points; 95% CI: − 1.00, − 0.22; *P* = 0.002). Moreover, dexmedetomidine-sufentanil reduced sufentanil consumption during the first 24 h (MD = -13.77 μg; 95% CI: − 18.56, − 8.97; *P* < 0.00001) and 48 h postoperatively (MD = -20.81 μg; 95% CI: − 28.20, − 13.42; *P* < 0.00001). Finally, dexmedetomidine-sufentanil improved patient satisfaction without increasing the incidence of side effects.

**Conclusions:**

Dexmedetomidine as an adjuvant to sufentanil for postoperative PCA can reduce postoperative pain score and sufentanil consumption.

## Background

Postoperative pain is a common complication after surgery. Notably, effective management of postoperative pain is a core aspect of enhanced recovery after surgery, it reduces hospital stay and overall hospital cost, while enhancing recovery and reducing mortality after surgery [1, 2]. Intravenous patient-controlled analgesia (PCA) is an effective method for management of postoperative pain, because variable pharmacokinetic and pharmacodynamic parameters among patients and drugs can benefit from individual titration [3]. Of the variety of drugs available for postoperative acute pain, opioids are regarded as the preferred treatment. However, opioid use can result in numerous side effects, including excessive sedation, nausea, vomiting, pruritus, constipation, and respiratory depression [4, 5]; therefore, it is important to provide opioid- sparing analgesia. Multimodal pain management has been recommended to enhance pain relief and reduce the side effects of postoperative PCA [6].

Dexmedetomidine is a highly selective α-2 adrenergic agonist that exhibits hypnotic, sedative, analgesic, and anxiolytic properties [7–9]. Importantly, it does not cause respiratory depression [9, 10]. Dexmedetomidine has been reported to reduce the incidence of postoperative cognitive dysfunction [11] and to improve postoperative sleep quality [12]. A previous meta-analysis [13] suggested that dexmedetomidine could reduce opioid consumption in postoperative PCA. However, many types of opioids were used for postoperative PCA in that analysis, and opioid consumption was calculated by opioid equianalgesic conversion, which could introduce clinical heterogeneity. Sufentanil is a widely used analgesic drug that provides more intense analgesia with extended duration and milder respiratory depression, compared to equivalent doses of fentanyl or morphine [14]. In recent years, there has been a gradual increase in the number of reports involving the use of sufentanil for intravenous PCA. Therefore, we performed a meta-analysis of randomized controlled trials (RCTs) to quantify the effect of dexmedetomidine as an adjuvant for postoperative PCA with sufentanil.

## Methods

This systematic review and meta-analysis was conducted in accordance with the recommendations of the Cochrane Handbook for Systematic Reviews of Interventions [15], and was reported in accordance with the Preferred Reporting Items for Systematic Reviews and Meta-Analyses (PRISMA) guidelines [16].

### Search strategy

PubMed, Embase, the Cochrane Library, and Web of Science were systematically and independently searched by 2 authors of this review, from the date of inception to January 12, 2018. The search strategy combined free text words and controlled vocabulary Medical Subject Heading terms, including “dexmedetomidine”, “sufentanil”, “sufentanil citrate”, “intravenous”, and “analgesia”; only English-language publications were included.

### Study inclusion and exclusion criterion

The eligible criteria were as follows: 1) Participants: adult surgical patients receiving postoperative intravenous PCA; 2) Intervention: dexmedetomidine- sufentanil for intravenous PCA; 3) Comparison: sufentanil alone for intravenous PCA; 4) Outcomes: at least 1 of the following outcomes—total sufentanil consumption, pain score, sedation score, patient satisfaction, sufentanil-related side effects (e.g., nausea, vomiting, pruritus, or respiratory depression), or dexmedetomidine- related side effects (e.g., hypotension and bradycardia); 5) Study design: only RCTs were included.

Exclusion criteria were as follows: 1) Use of sufentanil combined with drugs other than dexmedetomidine for postoperative PCA; 2) Use of opioids other than sufentanil for postoperative analgesia; 3) Intraoperative use of dexmedetomidine alone, rather than in combination with sufentanil for PCA after surgery; 4) Lack of specific outcomes reported within the trial; 5) Trials reported in retrospective studies, scientific meetings, correspondence, case reports, or review papers.

### Data extraction

The 2 reviewers independently extracted the following data from the included studies: first author’s name; publication year; country; number of patients in each group; type of surgery and anesthesia; and doses of dexmedetomidine and sufentanil in postoperative intravenous PCA. Primary outcomes were: 1) Pain intensity at 24 and 48 h postoperatively; and 2) Total sufentanil consumption during the first 24 and 48 h postoperatively. Secondary outcomes were: 1) Sedation score at 1 h postoperatively; 2) Incidences of nausea, vomiting, pruritus, and respiratory depression; 3) Number of patients satisfied with intravenous PCA; and 4) Incidences of hypotension and bradycardia. Authors were contacted to obtain additional information, if necessary. Regarding data extraction, any disputes were resolved by discussion with a third reviewer.

### Quality assessment

The 2 authors who performed searching and data extraction then independently read all included studies and evaluated the quality with the Cochrane risk of bias tool [17]. The following 7 items were assessed: random sequence generation (selection bias), allocation concealment (selection bias), blinding of participants and personnel (performance bias), blinding of outcome assessment (detection bias), incomplete outcome data (attrition bias), selective reporting (reporting bias), and other potential biases [17]. Each item was graded as “low risk of bias”, “unclear risk of bias”, or “high risk of bias”. If there was a dispute involving quality assessment, a consensus was reached by discussion with the third reviewer.

### Quality of evidence assessment

The grading of recommendations, assessment, development, and evaluation (GRADE) methodology [18] was used to evaluate the quality of evidence with 4 levels (high, moderate, low, and very low). Assessment items included the risk of bias, inconsistency, indirectness, imprecision, and publication bias. GRADE Pro software (GRADEpro, version 3.6) was used to perform assessments for all outcomes.

### Statistical analysis

Quantitative analysis was performed using Review Manager 5.3 (The Nordic Cochrane Centre for The Cochrane Collaboration, Copenhagen, Denmark). Pain intensity was assessed using a visual analogue scale (VAS) (0 to 10) or a numerical rating scale (NRS) (0 to 10; 0 indicated “no pain” and 10 indicated the “the worst imaginable pain”). NRS scores (0 to 10) were converted to VAS scores (0 to 10) [19]. For continuous data, when studies used median and interquartile range, these data were converted to mean and standard deviation, following an established protocol [20]. For dichotomous data, we calculated the risk ratio (RR) and 95% confidence interval (CI) by the Mantel-Haenszel method. For continuous data, when measuring methods were different, the standardized mean difference (SMD) with 95% CI was calculated; otherwise, the mean difference (MD) with 95% CI was calculated. Statistical heterogeneity was assessed by using the Q and I^2^statistics. *P* > 0.1 and I^2^ < 50% indicated a low level of heterogeneity among studies; for these, a fixed effects model was used. *P* < 0.1 and I^2^ > 50% indicated a high level of heterogeneity among studies; for these, a random effects model was used. Due to the limited number (< 10) of included studies, publication bias was not evaluated. Sensitivity analysis was performed by excluding each respective study from the pooled results to identify the source of heterogeneity [21] and assess the robustness of the results [22].

## Results

### Study selection and characteristics of studies

**Fig. 1:**
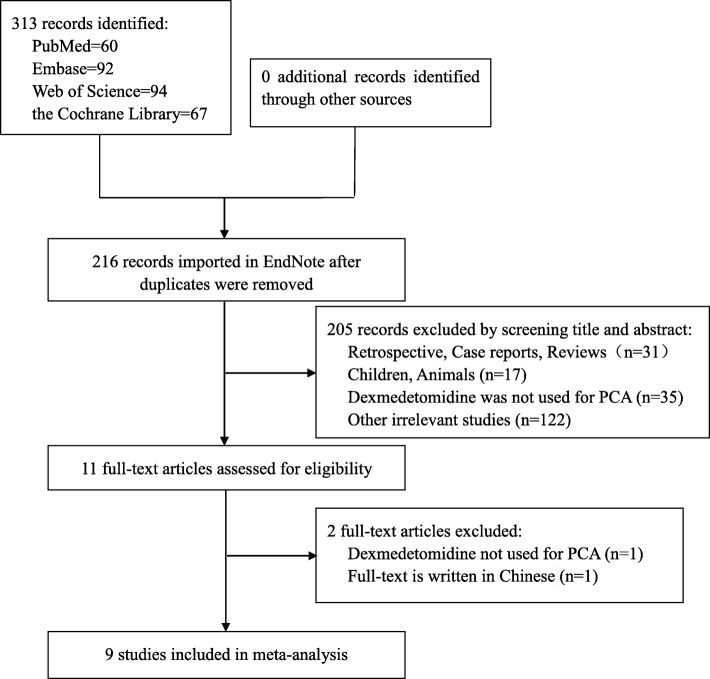
Study flow chart

A flow diagram of the literature search and evaluation is shown in Fig. 1. A total of 313 records were identified during the initial search (PubMed = 60, Embase = 92, Web of Science = 94, and Cochrane Library = 67). Ninety-seven records were excluded due to duplication; 205 were excluded because they did not meet the inclusion criteria upon screening of their titles and abstracts. The remaining 11 publications were screened by reading the full text. One article [23] was excluded because dexmedetomidine for intraoperative anesthesia, rather than for postoperative PCA. One article [24] was excluded because only the abstract was provided in English; another article was excluded because it described an ongoingstudy and only provided a summary. Finally, 9 RCTs [25–33] were included in this meta-analysis.

All eligible studies were published during the period from 2014 to 2018; in total, 907 patients were included in this analysis. The main characteristics of the included trials are shown in Table 1, and the PCA protocols are shown in Table 2.

**Table 1:** Characteristics of Included Trials

Trials (year)	Country	Group	Surgery	Anesthesia	Intraoperative Analgesia	Outcomes
Chen 2017 [25]	China	Control(*n* = 29) DEX(*n* = 30)	abdominal hysterectomy	general anesthesia	remifentanil	1,2,3,4,5
Gao 2018 [26]	China	Control(*n* = 101) DEX(*n* = 102)	abdominal operation	general anesthesia	Not specified	1,2,3,6,7,8,9,10
Nie 2014 [27]	China	Control (*n* = 38) DEX(*n* = 38)	caesarean section	spinal anesthesia	bupivacaine bupivacaine +DEX	1,3,6,8,9,10,11
Dong 2017 [28]	China	Control(*n* = 30) DEX(*n* = 30)	thoracotomy operation	general anesthesia	sufentanil	1,2,3,4,6,7,8,9,10, 11
Qin 2017 [29]	China	Control(*n* = 29) DEX(*n* = 29)	partial laryngectomy	general anesthesia	sufentanil+DEX	1,3,6,7,8,9,10, 11
Lu 2017 [30]	China	Control(*n* = 76) DEX(*n* = 75)	shoulder arthroscopy	general anesthesia+ brachial plexus block	DEX+ ropivacaine+ remifentanil	1,2,3,5,6,7,9, 11
Ren 2015 [31]	China	Control(*n* = 41) DEX1(*n* = 41) DEX 2 (*n* = 43)	thoracic surgery	general anesthesia	DEX + sufentanil	1,2,3,4,5,6,7,11
Ren 2015 [32]	China	Control(*n* = 27) DEX1(*n* = 28) DEX 2 (*n* = 27)	abdominal hysterectomy	general anesthesia	sufentanil DEX + sufentanil DEX + sufentanil	1,2,3,4,5,6,7,9,11
Xin 2017 [33]	China	Control(*n* = 47) DEX(*n* = 46)	laparotomy surgery	general anesthesia	remifentanil	1,2,6,8,9,10,11

1. pain scores at 24 h postoperatively; 2. pain scores at 48 h postoperatively; 3. sufentanil consumption during the first 24 h postoperatively; 4. sufentanil consumption during the first 48 h postoperatively;5. sedation score at 1 h postoperatively; 6. the incidence of PONV; 7. the incidence of pruritus; 8. patient satisfaction; 9. the incidence of bradycardia; 10. the incidence of hypotension; 11. the incidenceof respiratory depression

*DEX=* dexmedetomidine, *PONV=* postoperative nausea and vomiting

**Table 2:** PCA Protocols

Trials	Group	PCA Background Infusion		Bolus Dose		Lockout Interval
		sufentanil	DEX	sufentanil	DEX	
Chen 2017 [25]	Control	0.02 μg kg^− 1^ h^− 1^	-	0.02 μg/kg	-	10 min
	DEX + sufentanil	0.02 μg kg^− 1^ h^− 1^	0.05 μg kg^− 1^ h^− 1^	0.02 μg/kg	0.05 μg/kg	10 min
Gao 2018 [26]	Control	2 μg/h	-	2 μg	-	5 min
	DEX + sufentanil	2 μg/h	4 μg/h	2 μg	4 μg	5 min
Nie 2014 [27]	Control	0.015 μg kg^− 1^ h^− 1^	-	0.023 μg/kg	-	8 min
	DEX + sufentanil	0.015 μg kg^− 1^ h^− 1^	0.045 μg kg^− 1^ h^− 1^	0.023 μg/kg	0.07 μg/kg	8 min
Dong 2017 [28]	Control	0.048 μg.kg^− 1^.h^− 1^	-	0.024 μg/kg	-	10 min
	DEX + sufentanil	0.048 μg.kg^− 1^.h^− 1^	0.064 μg.kg^− 1^.h^− 1^	0.024 μg/kg	0.032 μg/kg	10 min
Qin 2017 [29]	Control	1.5 μg/h	-	1.5 μg	-	10 min
	DEX + sufentanil	1.5 μg/h	6 μg/h	1.5 μg	6 μg	10 min
Lu 2017 [30]	Control	0.04 μg kg^− 1^ h^− 1^	-	0.03 μg/kg	-	5 min
	DEX + sufentanil	0.04 μg kg^− 1^ h^− 1^	0.06 μg kg^− 1^ h^− 1^	0.03 μg/kg	0.045 μg/kg	5 min
Ren 2015 [31]	Control	0.02 μg kg^− 1^ h^− 1^	-	0.02 μg/kg	-	5 min
	DEX + sufentanil 1	0.02 μg kg^− 1^ h^− 1^	0.02 μg kg^− 1^ h^− 1^	0.02 μg/kg	0.02 μg/kg	5 min
	DEX + sufentanil 2	0.02 μg kg^− 1^ h^− 1^	0.04 μg kg^− 1^ h^− 1^	0.02 μg/kg	0.04 μg/kg	5 min
Ren 2015 [32]	Control	0.02 μg kg^− 1^ h^− 1^	-	0.02 μg/kg	-	8 min
	DEX + sufentanil 1	0.02 μg kg^− 1^ h^− 1^	0.02 μg kg^− 1^ h^− 1^	0.02 μg/kg	0.02 μg/kg	8 min
	DEX + sufentanil 2	0.02 μg kg^− 1^ h^− 1^	0.05 μg kg^− 1^ h^− 1^	0.02 μg/kg	0.05 μg/kg	8 min
Xin 2017 [33]	Control	0.04 μg kg^− 1^ h^− 1^	-	0.01 μg/kg	-	15 min
	DEX + sufentanil	0.02 μg kg^− 1^ h^− 1^	0.04 μg kg^− 1^ h^− 1^	0.005 μg/kg	0.01 μg/kg	15 min

*DEX=* dexmedetomidine

### Risk of Bias assessment

**Fig. 2:**
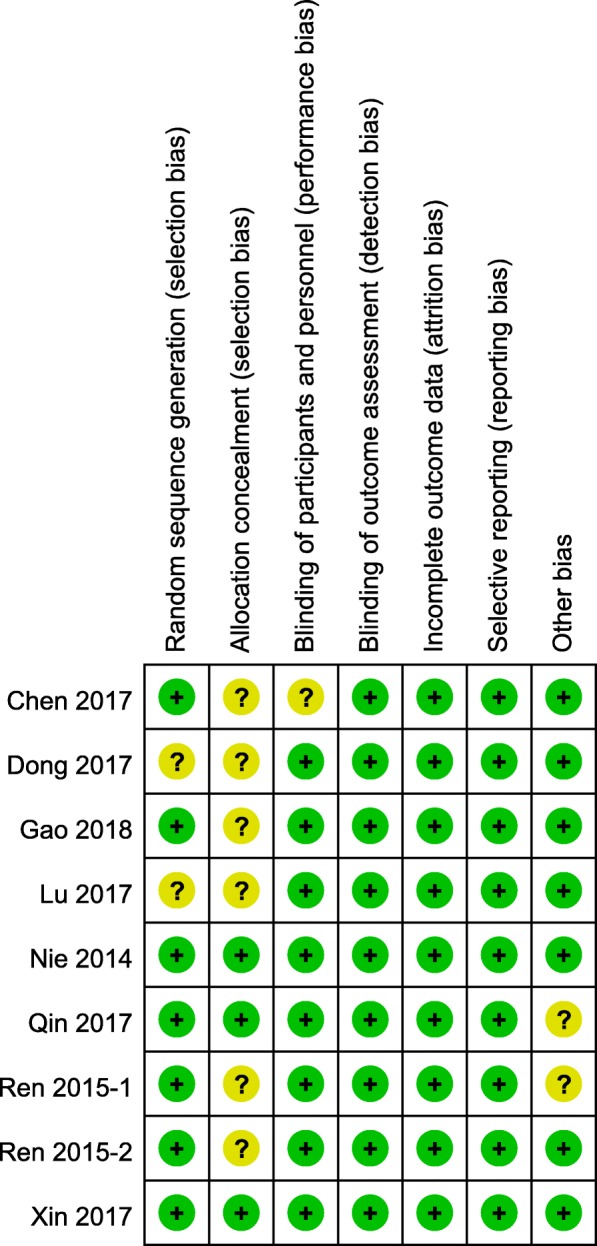
Risk of bias of the included studies, based on the Cochrane risk of bias tool

The details of methodologic quality are shown in Fig. 2. Two studies [28, 30] did not describe the details of random sequence generation. One study [25] showed an unclear risk, whereas the remaining 8 studies were judged to be low-risk with respect to blinding of participants and personnel. Two studies [29, 31] solely included male patients; thus, these studies had an unclear risk of other bias. Three studies [27, 29, 33] provided detailed descriptions of the methods of allocation concealment. All studies had low risks of bias due to blinding of outcome assessment, incomplete outcome data, and selective reporting.

### Results of meta-analysis

#### VAS score at 24 h postoperatively

**Fig. 3:**
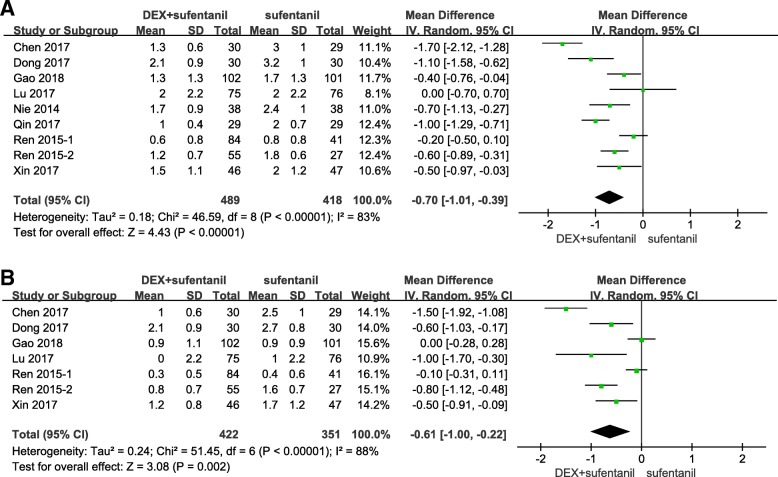
Forest plot of meta-analysis of VAS at 24 h (**a**) and 48 h postoperatively (**b**). DEX = dexmedetomidine; CI = confidence interval; VAS = visual analog scale

Nine studies reported pain intensity at 24 h postoperatively. I^2^ was 83%, which indicated high heterogeneity among the included studies. The pooled results indicated that patients receiving postoperative PCA with dexmedetomidine-sufentanil combination exhibited a significant reduction in pain intensity at 24 h postoperatively, compared with patients receiving sufentanil alone (MD = -0.70 points; 95% CI: − 1.01, − 0.39; *P* < 0.00001, Fig. 3a).

#### VAS score at 48 h postoperatively

Seven studies reported pain intensity at 48 h postoperatively. I^2^ was 88%, which indicated high heterogeneity among the included studies. Compared with patients receiving sufentanil alone for postoperative PCA, patients receiving dexmedetomidine-sufentanil combination for postoperative PCA exhibited a significant reduction in pain scores at 48 h postoperatively (MD = -0.61 points; 95% CI: − 1.00, − 0.22; *P* = 0.002, Fig. 3b).

#### Total sufentanil consumption during the first 24 h postoperatively

**Fig. 4:**
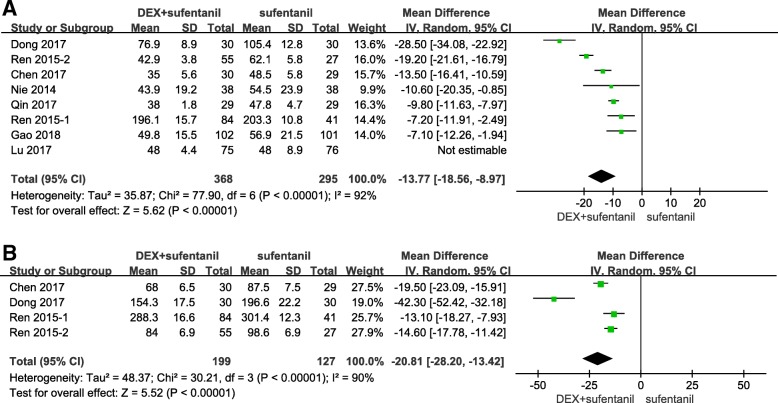
Forest plot of meta-analysis of sufentanil consumption during the first 24 h (**a**) and 48 h postoperatively (**b**). DEX = dexmedetomidine; CI = confidence interval

Eight studies reported total sufentanil consumption during the first 24 h postoperatively. The study by Lu et al. [30] measured sufentanil consumption in milliliters (ml) with an unclear concentration; other studies measured sufentanil consumption in micrograms (μg). After removing the study by Lu et al., the MD with 95% CI was calculated. I^2^ was 92%, which indicated high heterogeneity among the included studies. The pooled results indicated that patients receiving dexmedetomidine-sufentanil combination for postoperative PCA exhibited a significant reduction in total sufentanil consumption at 24 h postoperatively, compared with patients receiving sufentanil alone (MD = -13.77 μg; 95% CI: − 18.56, − 8.97; *P* < 0.00001, Fig. 4a).

#### Total sufentanil consumption during the first 48 h postoperatively

Four studies reported the total sufentanil consumption during the first 48 h postoperatively. I^2^was 90%, which indicated high heterogeneity among the included studies. The pooled results suggested that the combination of dexmedetomidine and sufentanil for PCA significantly reduced sufentanil consumption during the first 48 h postoperatively, compared with sufentanil alone (MD = -20.81 μg; 95% CI: − 28.20, − 13.42; *P* < 0.00001, Fig. 4b).

#### Sedation score at 1 h postoperatively

**Fig. 5:**
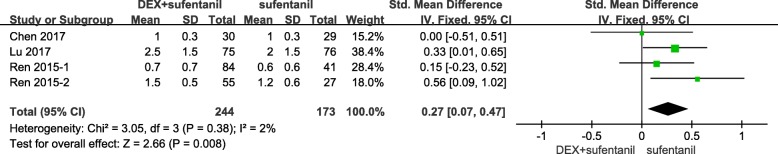
Forest plot of meta-analysis of sedation score at 1 h postoperatively. DEX = dexmedetomidine; CI = confidence interval

Four studies reported the sedation score at 1 h postoperatively. I^2^ was 2%, which indicated low heterogeneity among the included studies. The results indicated that patients receiving postoperative PCA with dexmedetomidine-sufentanil combination exhibited higher sedation scores at 1 h postoperatively, compared with patients receiving sufentanil alone (SMD = 0.27; 95% CI: 0.07, 0.47; *P* = 0.008, Fig. 5). Sensitivity analysis showed no significant differences between the two groups upon removal of the trials of Lu et al. [30] or Ren et al. [32], which indicated inconsistent results.

#### Sufentanil-related adverse events

**Fig. 6:**
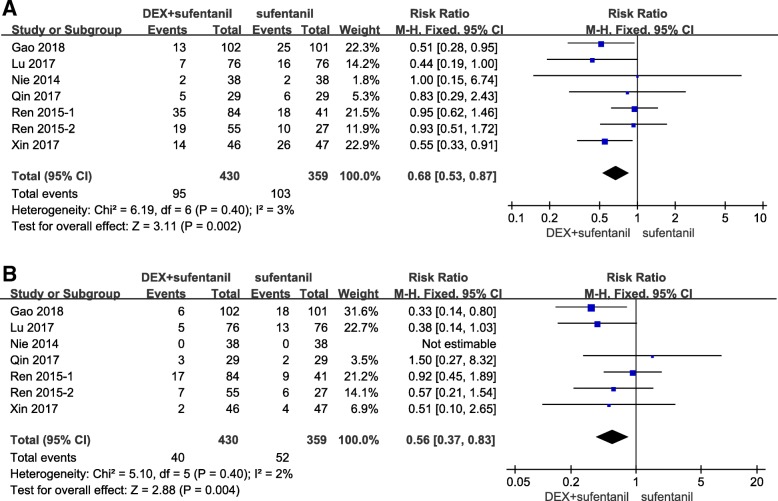
Forest plot of meta-analysis of postoperative nausea (**a**) and vomiting (**b**). DEX = dexmedetomidine; CI = confidence interval

Eight studies described the incidences of nausea, vomiting, and pruritus. Compared with patients receiving sufentanil alone, there were lower incidences of postoperative nausea (RR = 0.68, 95% CI: 0.53, 0.87; *P* = 0.002, I^2^ = 3%, Fig. 6a), vomiting (RR = 0.56, 95% CI: 0.37, 0.83; *P* = 0.004, I^2^ = 2%, Fig. 6b), and pruritus (RR = 0.54, 95% CI: 0.34, 0.83; *P* = 0.006, I^2^ = 0%) in patients receiving dexmedetomidine- sufentanil combination for postoperative PCA. Only 1 study [28] reported the incidence of respiratory depression (respiratory rate < 8beats per minute, lasting for more than 10 min); it found no significant difference between the dexmedetomidine- sufentanil and sufentanil groups.

#### Patient satisfaction

**Fig. 7:**
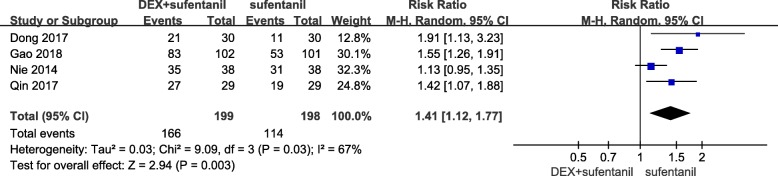
Forest plot of meta-analysis of patient satisfaction. DEX = dexmedetomidine; CI = confidence interval

Four studies reported the number of patients who were satisfied with intravenous PCA. I^2^ was 67%, which indicated high heterogeneity in the included studies. Patients receiving postoperative intravenous PCA with dexmedetomidine- sufentanil combination exhibited higher satisfaction than those receiving sufentanil alone (RR = 1.41, 95% CI: 1.12, 1.77; *P* = 0.003, Fig. 7).

#### Other outcomes

No significant differences were observed in the incidences of hypotension (RR = 1.39, 95% CI: 0.28, 6.93; *P* = 0.69, I^2^ = 30%) or bradycardia (RR = 1.83, 95% CI: 0.81, 4.15; *P* = 0.15, I^2^ = 0%) between the 2 groups.

### GRADE assessment

The qualities of evidence according to the GRADE approach are shown in Table 3. The GRADE level of evidence was very low for total sufentanil consumption during the first 24 and 48 h postoperatively, as well as for VAS scores at 24 and 48 h postoperatively. The GRADE level of evidence was low for sedation score at 1 h postoperatively, whereas it was moderate for patient satisfaction. The GRADE levels of evidence were high for the incidences of postoperative nausea, vomiting, pruritus, hypotension, and bradycardia.

**Table 3:** The Quality of Evidences

Outcome	MD/RR (95%CI)	Number of Participants (studies)	Quality of the evidence (GRADE)	Comments
VAS score at 24 h postoperatively	MD −0.70 [−1.01, − 0.39]	907(9 studies)	⊕ΟΟΟ Very Low	I^2^ statistic shows high level of heterogeneity at 83%, when studies used median and interquartile range, we converted these to mean and standard deviation (SD). We downgraded the quality of evidence for inconsistency and indirectness.
VAS score at 48 h postoperatively	MD −0.61 [−1.00, − 0.22]	773(7 studies)	⊕ΟΟΟ Very Low	I^2^ statistic shows high level of heterogeneity at 88% and when studies used median and interquartile range, we converted these to mean and standard deviation (SD). We downgraded the quality of evidence for inconsistency, indirectness.
Total sufentanil consumption during the first 24 h postoperatively	MD −13.77 [− 18.56, − 8.97]	663(7 studies)	⊕ΟΟΟ Very Low	I^2^ statistic shows high level of heterogeneity at 92% and part data were extracted from figures. We downgraded the quality of evidence for inconsistency, indirectness.
Total sufentanil consumption during the first 48 h postoperatively	MD − 20.81[− 28.20,-13.42]	326(4 studies)	⊕ΟΟΟ Very Low	I^2^ statistic shows high level of heterogeneity at 90% and part data were extracted from figures. We downgraded the quality of evidence for inconsistency, indirectness.
Sedation score at 1 h postoperatively	–	417(4 studies)	⊕⊕ΟΟ Low	SMD0.27 [0.07, 0.47] When studies used median and interquartile range, we converted these to mean and standard deviation (SD) and part data were extracted from figures. We downgraded the quality of evidence for indirectness and inconsistency.
Postoperative nausea	RR 0.68 [0.53, 0.87]	789(7 studies)	⊕⊕⊕⊕ High	
Postoperative vomiting	RR 0.56 [0.37, 0.83]	789(7 studies)	⊕⊕⊕⊕ High	
Pruritus	RR 0.54 [0.34, 0.83]	680(6 studies)	⊕⊕⊕⊕ High	
Patients’ satisfaction	RR 1.41 [1.12, 1.77]	397(4 studies)	⊕⊕⊕Ο Moderate	I^2^ statistic shows heterogeneity at 67%. We downgraded the quality of evidence for inconsistency.
Hypotension	RR 1.39 [0.28, 6.93]	490(5 studies)	⊕⊕⊕⊕ High	
Bradycardia	RR 1.83 [0.81, 4.15]	723(7 studies)	⊕⊕⊕⊕ High	

## Discussion

In this meta-analysis, we quantified the effect of dexmedetomidine as an adjuvant to sufentanil for PCA and found that dexmedetomidine improved postoperative pain intensity and reduced total sufentanil consumption. Furthermore, sufentanil-related side effects (e.g., postoperative nausea, vomiting, and pruritus) were reduced in the dexmedetomidine-sufentanil group; the incidences of dexmedetomidine-associated side effects (e.g., bradycardia and hypotension) did not increase.

In the past few decades, intravenous PCA has been commonly used for postoperative analgesia [34, 35]. Sufentanil is commonly used for the treatment of moderate to severe postoperative pain; however, the risk of adverse effects limits its use as a single method to manage postoperative pain [36–38]. Dexmedetomidine achieves ananalgesic effect by activation of α-2 adrenoceptors, thereby acting in a manner that differs from sufentanil; notably, combination of these drugs produces a synergistic analgesic effect without increasing the risk of respiratory depression [39].

Sufentanil-related complications were significantly reduced, while patient satisfaction was improved in the dexmedetomidine-sufentanil group, compared to the sufentanil group. These changes may be explained as follows: 1) Patients receiving dexmedetomidine-sufentanil combination for PCA used lower doses of sufentanil; and 2) Dexmedetomidine can decrease noradrenergic activity by inhibiting presynaptic α2 receptors in the locus coeruleus, or by reducing sympathetic outflow, which may induce postoperative nausea and vomiting [40].

With respect to the safety characteristics involved in the addition of dexmedetomidine to postoperative intravenous PCA, hypotension and bradycardia have been identified as the primary concerns [41]. In particular, for patients with stroke or coronary disease, the hypotensive or bradycardic actions of dexmedetomidine may be harmful. Upon administration of a high dose or rapid intravenous injection, dexmedetomidine produces hypertension by activating α-2 adrenoceptors on smooth muscle cells. When administered at clinically recommended concentrations, dexmedetomidine produces dose-dependent hypotension and bradycardia, due to the inhibition of neurotransmission in sympathetic nerves and reduction of sympathetic tone; this effect may also be mediated by the baroreceptor reflex and enhanced vagal activity [9, 10, 42]. In the present study, pooled results demonstrated no significant differences in the incidences of hypotension or bradycardia between the dexmedetomidine-sufentanil and sufentanil group; this might be a result of the small dose of dexmedetomidine used in these studies. Although no statistically significant difference was detected, there remains considerable concern with respect to the potential risks of hypotension and severe bradycardia associated with the use of dexmedetomidine. Moreover, dexmedetomidine could inhibit the release of corticosterone in response to adrenocorticotropic hormone stimulation after prolonged use or high dosage [43]. Enomoto et al. [44] reported that long-term dexmedetomidine administration might cause tolerance in infants, but there have been no reports of long-term use of dexmedetomidine for PCA in adults, likely because PCA is typically used for 2–3 days after surgery. Nonetheless, rebound hypertension and tachycardia after abrupt cessation of dexmedetomidine, as well as changes in tolerance and the potential for withdrawal syndrome, remain concerns when using dexmedetomidine.

The pooled results indicated that, regardless of the type of sedation score used, patients receiving postoperative intravenous PCA with dexmedetomidine-sufentanil combination exhibited higher sedation scores at 1 h postoperatively, compared with patients receiving sufentanil alone. However, interpretation of this result requires caution, because sensitivity analysis showed that there was no significant difference between the 2 groups. Notably, no oversedation events were reported in the included studies. Thus, the clinical significance of the outcome regarding postoperative sedation scores is unclear and further investigation is needed.

Peng et al. [13] found that patients receiving an opioid-dexmedetomidine combination for postoperative PCA experienced significantly greater pain relief and had significantly lower postoperative opioid consumption during the first 24 h postoperatively, compared with those receiving opioid alone. The results of the current meta-analysis were consistent with those findings, and provided evidence to support the safety of dexmedetomidine administration for more than 24 h. An updated meta-analysis by Peng et al. [45] also examined the safety of prolonged use of dexmedetomidine after surgery; our present findings are consistent with those of the updated analysis. However, previous meta-analyses included studies that used various opioids and were published before 2017. Although data regarding opioid equianalgesic conversion factors have been previously published, their pharmacokinetics and pharmacodynamics were not exactly same. In our meta-analysis, we solely included studies using sufentanil, which has a smaller volume of distribution, shorter elimination half-life, and more rapid recovery than either fentanyl or morphine [46]. Sufentanil exhibits a wider therapeutic index than other opioids for PCA [28], is the most potent available analgesic, and is the most commonly used for intravenous PCA. Six of 9 RCTs in our meta-analysis were published after 2017. When dexmedetomidine was added to PCA, previous meta-analysis [45] reported that the morphine-equivalent consumptions during the first 24 and 48 h after surgery decreased by 12.16 mg and 10.15 mg, respectively. This suggested that dexmedetomidine may be ineffective during the first 24 to 48 h after surgery. In contrast, our results showed that when dexmedetomidine was added to PCA, sufentanil consumption during the first 24 and 48 h postoperatively decreased by 13.77 μg and 20.81 μg, respectively. Our meta-analysis therefore indicated that the analgesic effect of dexmedetomidine continued throughout the first 48 h postoperatively.

There were several limitations in our meta-analysis. First, it included a small number of studies; however, we included all literature available. Second, all studies investigated Chinese adult patients, although they were reported in English. It remains unknown whether our findings are applicable to patients of other ethnicities. Third, surgery types and perioperative anesthesia protocols varied among studies, as did the doses of dexmedetomidine and sufentanil; thus, the included studies exhibited high heterogeneity. Fourth, the present study did not assess the dose-response effects for different types of surgeries. Additional RCTs are needed to identify the optimal doses of dexmedetomidine and sufentanil for different surgeries. Finally, publication biases and potential biases may influence our results.

## Conclusions

Compared with sufentanil alone, dexmedetomidine- sufentanil combination for postoperative intravenous PCA may achieve better analgesia and patient satisfaction, thereby reducing sufentanil consumption and sufentanil-related complications.

## Abbreviations

CI: Confidence interval
DEX: Dexmedetomidine
GRADE: The grading of recommendations, assessment, development, and evaluation methodology
MD: Mean difference
NRS: Numerical rating scale
PCA: Patient-controlled analgesia
PONV: Postoperative nausea and vomiting
PRISMA: Preferred reporting items for systematic reviews and meta-analyses
RCT: Randomized controlled trial
RR: Risk ratio
SMD: Standardized mean difference
VAS: Visual analogue scale
